# Resection of petroclival clear cell meningioma by the anterior transpetrosal approach: diagnosis of rare pathology and improvement of preoperative hearing disturbance

**DOI:** 10.3171/2022.1.FOCVID21219

**Published:** 2022-04-01

**Authors:** Ken Matsushima, Michihiro Kohno, Nobuyuki Nakajima, Norio Ichimasu, Sho Onodera, Jiro Akimoto

**Affiliations:** Department of Neurosurgery, Tokyo Medical University, Tokyo, Japan

**Keywords:** cerebellopontine angle tumor, high-grade meningioma, magnetic resonance imaging, posterior fossa, skull base surgery, video

## Abstract

Clear cell meningioma is a rare histological variant of meningioma, which often recurs aggressively. This video demonstrates a patient with a petroclival clear cell meningioma, which was resected completely through the anterior transpetrosal approach. The absence of intratumoral spotty signal voids on preoperative susceptibility-weighted imaging (SWI) suggested that the tumor was a meningioma rather than a schwannoma, although typical imaging features of meningioma were not observed. After surgery, the patient’s preoperative hearing disturbance improved from class D to class A, which the authors had sometimes experienced in cerebellopontine angle meningioma surgeries. Careful observation over a 2.5-year period revealed no tumor recurrence, without additional treatment.

The video can be found here: https://stream.cadmore.media/r10.3171/2022.1.FOCVID21219

## Transcript

This video shows the preoperative image characteristics, microsurgical resection, and postoperative hearing improvement of a patient with petroclival clear cell meningioma who was treated by the anterior transpetrosal approach.

### 0:36 Clinical History.

The patient was a 28-year-old woman who presented with progressive hearing disturbance and tinnitus. Neurological examination revealed slight facial sensory impairment.

### 0:48 Preoperative Radiological Images.

MRI displayed a right petroclival tumor compressing the brainstem and extending into the Meckel’s cave. CT displayed slight bone erosion around the Meckel’s cave. Angiography did not detect any tumor stains.

The lesion was isointense on T1-weighted images and hyperintense on T2-weighted images, and heterogeneous enhancement was displayed on contrast-enhanced T1-weighted images. It looked like the trigeminal schwannoma, but intratumoral spotty signal voids were not observed on SWI, suggesting meningioma rather than schwannoma.

As in this present case, some cerebellopontine angle meningiomas can mimic schwannomas, owing to the lack of its typical imaging features, such as the dural tail sign, intratumoral calcification, and hyperostosis. In such cases, the absence of intratumoral spotty signal voids on SWI is useful to distinguish meningiomas from schwannomas.^1^

### 1:51 Setup and Exposure.

The anterior transpetrosal approach was planned with the aim of complete tumor removal under SEP, MEP, ABR, and electromyography of the extraocular, masseter, and facial muscles.

After placing the lumbar drainage, the patient was put in the park-bench position, and a question-mark skin incision was made. For dural closure, a temporal subgaleal flap was made using the loose areolar tissue while preserving the temporal fascia. The usefulness of the areolar tissue graft was recently emphasized for plastic and skull base surgeries. Its rich vascular plexus enables early engraftment and long-term graft survival. Temporal craniotomy was performed, and the temporal base dura was peeled from the middle skull base. After cutting the middle meningeal artery and dissecting the greater superficial petrosal nerve, V3 dissection was started from the foramen ovale. The petrous apex was exposed, and the so-called Kawase’s triangle was drilled out.

After the anterior petrosectomy, we incised the temporal dura, ligated the superior petrosal sinus, opened the posterior fossa dura, and cut the tentorium while paying attention not to injure the trochlear nerve. Then, the lateral wall of the Meckel’s cave was opened to expose the tumor.

### 3:20 Lesion Removal.

The tumor was soft and yellowish, without significant bleeding, mimicking a schwannoma. We carefully dissected the displaced trigeminal nerve and confirmed the petrous portion of the internal carotid artery using an ultrasonic Doppler. Epiarachnoid dissection was performed along the ventral wall of the tumor, and the contralateral abducens nerve was identified. On the cranial side, the contralateral oculomotor nerve was meticulously dissected, exposing the left PCA and SCA. The intraoperative pathological diagnosis was clear cell meningioma. The thinned right abducens nerve was identified and carefully preserved. The internal acoustic meatus was opened by additional osseous drilling, to achieve a sufficient operative field and to remove the intrameatal tumor. By continuing the epiarachnoid dissection, the basilar artery and its top, bilateral PCA, and right SCA were exposed. The facial and vestibulocochlear nerves were displaced caudally together with the trigeminal nerve. The dural attachment was not present, as mentioned in previous reports.^2^ Gross-total resection of the tumor was achieved while preserving all the exposed cranial nerves and major vessels, owing to the soft tumor and the absence of the dural attachment.

Bone wax was used to cover the bone defect in the middle cranial fossa, and the dura was closed using the temporal subgaleal flap. We placed back the craniotomy flap and closed the temporal muscle and skin flap in a watertight fashion.

### 5:00 Postoperative Course.

The patient developed slight diplopia after the surgery, but her hearing disturbance improved from class D before the surgery to class A after the surgery, as we had experienced in several meningioma patients.^3^ Her tinnitus also disappeared after the surgery.

Postoperative CT displayed the extent of osseous drilling around the petrous apex. The pathological diagnosis was clear cell meningioma, same as the intraoperative consultation results. Contrast-enhanced MRI at 30 months’ follow-up confirmed complete resection of the lesion and no tumor recurrence without any additional treatment.

## Acknowledgments

We thank Ms. Eriko Hikawa and Ms. Yuri Miyazaki for their assistance in preparing this video.

## Disclosures

The authors report no conflict of interest concerning the materials or methods used in this study or the findings specified in this publication.

## Author Contributions

Primary surgeon: Kohno. Assistant surgeon: Matsushima, Nakajima, Ichimasu, Onodera. Editing and drafting the video and abstract: Matsushima. Critically revising the work: Kohno. Reviewed submitted version of the work: Kohno, Nakajima. Approved the final version of the work on behalf of all authors: Kohno. Supervision: Akimoto.

## Supplemental Information

### Previous Presentations

Preliminary data from this publication were presented virtually at Surgeon’s Log, a webinar hosted by Global Brainsurgery Initiative and the North American Skull Base Society in July 2021.
